# TwiC or treat? Are trials within cohorts ethically defensible?

**DOI:** 10.1177/1740774517746622

**Published:** 2017-12-17

**Authors:** Charles Weijer, Cory E Goldstein, Monica Taljaard

**Affiliations:** 1 Rotman Institute of Philosophy, Western University, London, ON, Canada; 2 Ottawa Hospital Research Institute, Ottawa, ON, Canada; 3 School of Epidemiology and Public Health, University of Ottawa, Ottawa, ON, Canada

Pragmatic randomized controlled trials (RCTs) seek to evaluate interventions in real-world conditions and, thereby, directly inform decisions made by patients, health providers and health system managers.^1^ The recent rise in novel methods to push trials toward being more pragmatic raises several complex issues. Two articles in this issue of *Clinical Trials* explore the ethical issues raised by one new design, called trials within cohorts (TwiCs).^2,3^ In this commentary, we critically evaluate the TwiC design, with an emphasis on informed consent.

Relton et al.^4^ first proposed the TwiC design to “recruit a greater quantity and more representative sample of patients.” The basic idea of TwiCs is to create a longitudinal cohort of patients to serve as a platform for the conduct of multiple RCTs. First, patients with a condition of interest are enrolled in a large longitudinal cohort in which their health data are collected from the electronic health record. Second, for each RCT, all eligible patients within the cohort are identified and a subset is randomly selected to be offered the study intervention. The outcomes of patients selected for the intervention group are compared to those of all remaining eligible patients in the cohort who receive usual care as determined by their physician. It is important to note that in the TwiC design the experimental intervention cannot be one that is routinely available to all members of the cohort. Furthermore, the control group cannot receive a protocolized intervention, even a drug that is routinely prescribed in practice. Finally, the outcomes of interest must be routinely collected outside the trial.

The article by Kim et al.^2^ offers a thoughtful analysis of the ethical issues raised by the TwiC design. The approach to consent originally used in the TwiC design involves no pre-randomization discussion of future RCTs. At the time of enrollment into the longitudinal cohort, patients are informed of data collection procedures but are not informed of “the possibility of future randomization and future contact for intervention studies.”^2^ Patients who are randomly selected for the intervention group in a subsequent RCT provide informed consent for that intervention; those in the control group provide no further informed consent, as they have already consented to the use of their data and usual medical care. Kim et al.^2^ argue that this approach may be ethical in some cases and maintain that “those not selected [for the intervention group] do not need to give consent for that random selection any more than individuals who are not selected in a random-digit-dial telephone survey need to give prior consent for randomization.” Informed consent is only required when “randomization leads to any potential alterations in the way subjects are treated.”^2^ Notwithstanding this argument, they admit that this approach is unlikely to fulfill regulatory requirements.

Given these “regulatory obstacles,” Kim et al.^2^ proffer a new approach involving pre-randomization broad consent. At the time of recruitment into the cohort, patients provide “specific consent for the cohort study and *also* broad consent.”^2^ Broad consent involves consent to participation in future RCTs, including “information about randomizations for future [RCTs], for future contact if randomized to the intervention arm … of [an RCT], and for use of their data in future [RCTs] if randomized to the control arm.”^2^ As noted above, only patients randomly selected for the intervention group provide consent for the study intervention. Thus, the authors assert, all participants will “have given informed consent to every aspect of their research participation in the TwiC” and no waiver of consent is required.^2^

In a second paper, Vickers et al.^3^ provide a detailed defense of this approach, which they call “just-in-time consent.” They point out that the usual informed consent process may “cause significant and persistent anxiety, distress and confusion in patients.”^3^ Patients receive information about interventions that they may not actually receive, which contributes to information overload and perhaps “disappointment about not receiving an appealing-sounding experimental intervention.”^3^ Just-in-time consent addresses these issues, we are told, by providing patients with the information they need only when they need it. Thus, the approach seeks to “reduce patient distress … and to enhance patient autonomy.”^3^ While there is, as yet, no evidence that just-in-time consent achieves these ends, the authors “propose research to determine the value” of this approach.^3^

In what follows, we make three points about the TwiC design. First, we argue that the original approach to informed consent for TwiCs is unethical and should not be used. Second, we acknowledge that the just-in-time consent model is preferable to the original, but argue that a waiver or modification of consent is nonetheless required. Third, we caution that TwiCs raise unique methodological issues which should be addressed in their design and analysis.

## The original approach to consent in TwiCs is unethical

The original approach to informed consent in TwiCs, involving no pre-randomization discussion of future RCTs, needs to be understood in light of the goals of recruiting “a greater quantity and more representative sample of patients.”^4^ Indeed, these goals will be achieved effectively if (1) all (or most) patients with the condition of interest enroll in the longitudinal cohort and (2) all (or most) eligible patients enroll in each RCT conducted within the cohort. Providing patients solely with information on data collection aspects is a means to achieve (1), and approaching only those patients randomly selected to the intervention group for consent (while enrolling all remaining patients in the control group without their consent) are means to achieve (2). Unfortunately, the proposal violates the ethical principle of respect for persons.

To understand the shortcomings of this approach, it is useful to recall the foundational ethical problem of human research ethics: what justifies exposing people to risk in research for the benefit of others? Part of the justification lies in treating individuals with the respect due to them as rational agents. The philosopher Immanuel Kant argued that this requires that rational agents be treated as ends in themselves and never as mere means to an end. Research participants are treated with respect when they are given a reasonably complete description of the research project and they identify the goals of the study as valuable to themselves. In an important sense, by providing their informed consent participants agree to take on the goals, or ends, of the project. In so doing, the ends pursued are not merely those of the researcher, they become the participants’ ends as well. While there are other possible theoretical groundings for consent, this framing helps us understand the importance of informed consent and the centrality of disclosing the aim or purpose of the study to the consent process.

This framing highlights several problems. To speak plainly, certain aspects of the TwiC design seem like a trick to increase RCT enrollment. By randomly selecting a subset of participants for the intervention group (rather than randomly allocating all participants to intervention or control groups), the design allows some participants (namely, those in the intervention group) to be presented with a simplified choice while others (namely, those in the control group) are presented with no choice at all. Doing so is wrong precisely because it treats participants as a mere means to an end, rather than as ends in themselves. To be fair, Kim and colleagues’ defense of the approach seems half-hearted. The comparison to “individuals who are not selected in a random-digit-dial telephone survey” is inapt as they are in no sense research participants; patients in the control group of an RCT plainly are research participants, because they are targets of the control intervention and their health data are used. As they are research participants, there is a presumption that their informed consent is required.

Because of the importance of informed consent, exceptions require a high threshold for permissibility. And, to be clear, this is a matter of the ethics of research, not merely one of regulation. The Council for International Organizations of Medical Sciences guidelines set out three requirements for a waiver or modification of consent that meet the ethical threshold: (1) the “research has important social value,” (2) the “research would not be feasible or practicable to carry out without the waiver or modification” and (3) the “research poses no more than minimal risks to participants.”^5^ As broad consent to participate in future RCTs could be employed in any TwiCs study, in no case would the research be infeasible or impracticable without the original approach to consent. Thus, a waiver of consent cannot be used to support the original approach to consent. Accordingly, we conclude that the original approach to consent in TwiCs is unethical and should not be used.

## Just-in-time consent requires a waiver or modification of consent

In their classic text, *A History and Theory of Informed Consent*, Faden and Beauchamp^6^ distinguish between consent as autonomous authorization and effective consent. Autonomous authorization is given when consent satisfies the ethical requirements of respect for persons, while effective consent requires only that policy provisions be fulfilled. Particularly in an environment in which regulatory compliance looms large, it is easy—and perhaps even tempting—to run these two distinct senses of consent together. But, we contend, this is an error as it is possible to satisfy one sense of consent while failing to satisfy the other.

We agree with Kim and colleagues that just-in-time consent is preferable to the original approach to consent for TwiCs. Patients are informed of the possibility of enrollment in unspecified future RCTs and are given an upfront opportunity to refuse. But does this approach fully satisfy the ethical requirements of informed consent? We think not. In their analysis, Kim et al.^2^ focus on the elements of research participation and, seeing that participants in the control group will have consented to random selection and use of their data, they conclude that their informed consent has been obtained. But even if participants have consented to each study intervention and data collection procedure, they have not provided autonomous authorization. For this, they must adopt the ends of the study as their own. If the purpose of a study is not disclosed specifically, participants cannot do this.

Indeed, the Council for International Organizations of Medical Sciences^5^ guidelines require that “information about the aims” of the study be disclosed to prospective participants. A similar requirement is found in the US Common Rule (see: 45 *Code of Federal Regulations* 46.116(a)(1)). The ethical framework provided above makes clear why this requirement must not be glossed over, or erroneously viewed as satisfied by a statement so vague as to encompass any study involving the disease in question. As just-in-time consent involves not disclosing the specific aims of the RCT to the control group, a modification of consent must be applied for and granted by a research ethics committee. Researchers will need to frame their justification for the modification of consent in terms of the social importance of the research, the infeasibility of proceeding without the modification and risk to participants.

This framework also casts into doubt Vickers et al.’s^3^ claim that just-in-time consent will “enhance patient autonomy.” While the approach may reduce patient anxiety or improve comprehension of the elements of consent, these ends may be achieved at the expense of the broader goal of autonomy.

## Methodological and practical implications of TwiCs must be addressed

While the TwiC design has distinct advantages for both internal and external validity,^7^ the extent to which these benefits are realized depends on adopting appropriate methods. Due to post-randomization consent in the intervention arm, the proportion of intervention arm participants who refuse or do not adhere will likely be greater than in a conventional pragmatic RCT. To avoid bias, the primary analysis should be based on intent to treat (or alternatively, utilize instrumental variables to estimate the causal effect of the intervention); to allow a true intent-to-treat analysis and realize the strengths of the TwiC design, data collection should indeed be complete. Power calculations should demonstrate how non-adherence and unequal allocation ratios have been taken into account, and effect size estimates should consider both the anticipated refusal rate and the expected association between the propensity to refuse and the outcome. Cohort sizes also should be adequate to allow repeated sampling of the required numbers of participants in the intervention group; if the size of the intervention group starts increasing relative to the remaining control arm participants, the design can lose efficiency.^8^

Finally, it is important to recognize the scope of application of the TwiC design, which is limited in several important respects. The design is most suited to stable populations of patients with a chronic condition, conditions for which many RCTs are likely to be conducted, and trials involving no blinding, treatment as usual in the control arm and outcome measures that can be assessed using data in the electronic health record.^4^ It is worth emphasizing that the control in envisioned RCTs cannot involve protocolized standard of care interventions, but must be usual care that is prescribed by the patient’s physician (compare with Vickers et al.^3^). The experimental intervention in envisioned RCTs also cannot be generally available to those in the control group, effectively ruling out the use of this design for head-to-head comparisons of usual care interventions.
